# Subjective social status and well-being of adolescents and young adults in Ghanaian schools: conditional process analysis

**DOI:** 10.1186/s40359-023-01158-7

**Published:** 2023-04-18

**Authors:** Frank Quansah, Edmond Kwesi Agormedah, John Elvis Hagan, James Boadu Frimpong, Francis Ankomah, Medina Srem-Sai, Kevin Dadaczynski, Orkan Okan, Thomas Schack

**Affiliations:** 1 Department of Educational Foundations, University of Education, Winneba, P. O. Box 25, Winneba, Ghana; 2 Department of Business & Social Sciences Education, University of Cape Coast, PMB Cape Coast, Cape Coast, Ghana; 3 Department of Health, Physical Education and Recreation, University of Cape Coast, PMB Cape Coast, Cape Coast, Ghana; 4 Neurocognition and Action-Biomechanics-Research Group, Faculty of Psychology and Sports Science, Bielefeld University, Postfach 10 01 31, 33501 Bielefeld, Germany; 5 Department of Education and Psychology, University of Cape Coast, PMB Cape Coast, Cape Coast, Ghana; 6 Department of Education, SDA College of Education, P. O. Box AS 18, Asokore-Koforidua, Ghana; 7 Department of Health, Physical Education, Recreation and Sports, University of Education, Winneba, P. O. Box 25, Winneba, Ghana; 8 Department of Health Science, Fulda University of Applied Sciences, 36037 Fulda, Germany; 9 Centre for Applied Health Science, Leuphana University Lueneburg, 21335 Lueneburg, Germany; 10 Department of Sports and Health Science, Technical University Munich, 80992 Munich, Germany

**Keywords:** Adolescent, Young adult, Monetary resource, Sense of coherence, Social status, Well-being

## Abstract

**Background:**

With the growing concern and interest in the mental health and well-being of adolescents and young adults (AYAs) including those in schools, many studies have explored the bivariate relationship between subjective social status (SSS) and AYAs’ subjective well-being (SWB). Acknowledging the spurious nature of this relationship, we assessed the relationship between SSS and SWB of AYAs in schools within Northern Ghana, focusing on the conditional indirect effect of monetary resource (MR) and sense of coherence (SoC).

**Methods:**

We utilised a cross-sectional descriptive design to survey 1096 senior high school students from two regions in Ghana’s Northern zone through a stratified sampling method. A questionnaire consisting of a number of calibrated standardized measures was used for the data collection. The data were processed using SPSS and PROCESS Macro and analysed using Hayes’ conditional process analysis.

**Results:**

The results revealed that students’ MR significantly moderated the relationships between SSS and SoC as well as SSS and SWB. A significant moderated mediation effect of MR and SoC on the relationship between SSS and SWB was found. Particularly, AYAs who reported higher levels of MRl, SSS and SoC reported a better SWB.

**Conclusion:**

The findings underscore the relevance of providing sufficient financial support for students in secondary schools in Ghana; thus, highlighting the sheer relevance of economic capital as a leading factor for better well-being. The findings also place much emphasis on building students’ personal coping mechanisms as a key variable in explaining how the students’ SSS and MR translate into having positive mental health outcomes.

## Introduction

Globally, mental health and well-being among adolescents and young adults (AYAs) in schools are of increasing concern to scholars and educators in health-related fields and beyond. Accordingly, positive psychologists have paid much attention to students’ psychological or subjective well-being (SWB) in the academic literature [1–3]. SWB reflects individuals’ cognitive (i.e., appraisal of life satisfaction) and affective (frequency of positive and negative affect) evaluations of their lives [4, 5]. Recent studies have discovered low levels of SWB among AYAs in schools or other educational settings [6–8]. For example, Dadaczynski et al. [6] in Germany and Dodd et al. [7] in Australia found low or very low levels of well-being for some cross-section of university students. Meanwhile, low levels of SWB among AYAs in school have been attributed to unsafe learning environments, family structure, financial distress, social structures, and other structural disparities [9–11]. Low SWB could negatively affect AYAs’ academic achievement, social capital development and engagement at the school and community levels [12].

Earlier research has illuminated the potential role of subjective social status (SSS) as a strong psychological factor, which, in turn, can positively influence SWB among AYAs in schools [13–15]. SSS is a comprehensive measure of one’s social position or perceived social standing relative to a given social group [16]. AYAs’ SSS reflects how they rank themselves relative to others in their school and community [17]. This social comparison is inevitable largely because it is an innate human desire to improve as stipulated by the social comparison theory [18, 19]. Among the AYAs, extant scholars have established the predictive power of SSS on various health and well-being outcomes [20, 21]. For example, in the USA, Niu et al. [22] found that a higher level of SSS is associated with better mental health among the youth. Also, in Germany, SSS was positively associated with mental health and SWB among university students [6, 23]. Related discoveries were reported in other regions such as the Americas [24–26], Europe [27–29], Asia [30–32] and Australia [33].

Although empirical studies have examined the relationship between SSS and SWB among AYAs, little attention has been paid to the roles of key variables such as sense of coherence (SoC) and monetary resource (MR) in this association. Arguably, this relationship between SSS and SWB among AYAs in schools can be explained by SoC and MR as either serving as a catalyst to strengthen or weaken the relationship. Thus, SoC was used as a mediating variable and MR was used as a moderating variable in this study. Grounded in the Antonovsky’s salutogenic theory, SoC is described as a global orientation that enables one to perceive events of the world or one’s life in general (e.g., school-related conditions) as comprehensible (i.e., feeling confident that the stimuli deriving from ones internal and external environments are ordered, structured, predictable and explicable rather than chaotic), manageable (i.e., feeling confident that one possesses the resources to meet the demands posed by these stimuli), and meaningful (i.e., feeling confident that these demands are challenges and worthy of the investment of time and energy and commitment) [34–36]. According to the theory, AYAs with strong SoC are better able to deal with the stressors of everyday life related to their academic and social status (i.e., subjective and objective SES), solve problems in positive ways more easily, and use the resources at their disposal to counter these stressors [34–38]. The AYAs in schools with a higher SoC level are likely to exhibit a better health status and SWB. Supporting the Antonovsky’s salutogenic theory, numerous studies have confirmed the association between SoC and mental health/SWB of AYAs in Europe [39–43], Asia [44] and Africa [45]. The Antonovsky’s salutogenic theory depicts that an individual’s SSS may determine his or her SoC (e.g., manageability - feeling self-assured that one possesses the required resources (e.g., monetary resources) to meet life demands or stressors) [35, 46, 47]. Thus, AYAs’ SSS is positively associated with their SoC [48]. It has been found that SSS affects individuals’ subjective sense of power and control in social encounters like academic stress [49, 50].

Drawing on the salutogenesis theory, students’ monetary resource (MR) - an element of generalized resistance resources [GRRs] - enhances their SoC [34–36, 51]. According to Eriksson and Lindström [52], students’ MR could be considered as part of the general resistance resources in SoC for mitigating potential life adversities. Hence, the adequacy of MR might be considered a key factor (i.e., a resistant resource) that could aid individuals in perceiving their lives in pursuit of positive health in a more consistent and organized way [46]. Thus, the strength of SoC depends on the availability of monetary resources possessed by the individual, family, and society. In this inquiry, students’ MR is conceptualised as money at their disposal. This money may include school fees, or money for buying food and school-related materials and could come from several sources such as parents/guardians, siblings, family relatives, teachers, friends or even money individuals have earned themselves [53–58]. When students have adequate MR, they have a better chance of dealing with life’s challenges, however, the absence of MR can become a stressor which may affect SoC and consequently, health outcomes [34–36]. It must be mentioned that adequate MR may also contribute to the perception that there are resources at one’s disposal to meet demands, thereby enhancing SoC and increasing life satisfaction. However, AYAs with low family income or financial resources may experience school and living conditions that are not conducive to the development of a sense of comprehensibility, manageability, and meaningfulness. Additionally, AYAs’ MR could influence how they rank or see themselves with others in the school and community [59] and their integration through participation in school and social activities with their peers, which in turn, can affect their SWB.

Though there are no studies directly linking students’ MR with SSS, SoC and SWB, there are studies that have found that income (an aspect of monetary resource) positively relates to SSS, SoC, and health outcomes in diverse populations [19, 29, 33, 60–63]. It has also been found that access to sufficient MR may provide opportunities for participation in activities that are perceived as meaningful, relevant, and enjoyable [19]. Supported by the self-determination theory, students’ MR (i.e., financial resources) might also contribute to their participation in social activities which would foster their social relatedness and autonomy (two of three basic psychological needs), while lack of MR might impede these psychological needs with potential negative effects on SWB [64, 65]. Obviously, a lack of MR can have a disturbing effect on students’ SWB, especially, in the African context where poverty continues to be on the rise. For instance, Kumsa et al. [66], in their study in Ethiopia, found that students had difficulties in obtaining money from their families or other funding sources which affected their ability to make social connections with peers in school and community. This financial situation might eventually affect their internal coping capacity (i.e., SoC), SSS and health outcomes.

### The present study

Ghana is a sub-Saharan African country with a medium-level human development index, placing her above the regional average. Poor socio-economic status among residents including AYAs is a major concern among stakeholders and policy makers. For example, the international poverty rate in Ghana as of 2021 was projected at 11.3%, with the same rate noted in 2022. This figure represented a slight increase compared to 2019 when the poverty rate was measured at 11.1% [67]. Again, a report from Ghana Statistical Service [GSS] indicated that Ghana has experienced a continuous reduction in poverty level over the years [68]. The population multidimensional poverty increases from about 13.6 million (44.1%) people to 14.4 million (46.7%). More than half of the population in nine regions (Ahafo, Western North, Bono East, Upper East, Upper West, Oti, Northern, Savannah, and North East) are multidimensionally poor, ranging from 53.0 to 77.6% [68]. Of great concern is the Northern region which saw its high level of poverty fall only marginally from 55.7 to 50.4%. The Northern region has seen the least progress in poverty reduction. This trend is a major issue for the country given that the Northern region now makes up the largest number of poor people in Ghana relative to other regions [69]. In the same report by GSS [68], nearly half (49.1%), representing 15.1 million of the population experience food insecurity. The prevalence of food insecurity is higher in rural areas than in urban areas. Households composed of dependant children and the elderly are more food insecure compared to those without any dependant. This poor socio-economic status may affect AYAs’ SSS, MR and SoC as well as SWB. Bull et al. [70], for instance, established that adolescents in the Northern region are happy when they have food in stock.

In the Ghanaian socio-economic space, AYAs are considered vulnerable as they face challenging circumstances and new experiences in their life with a myriad of mental health problems (e.g., anxiety, depression and sleeping disorders) [71–74]. They are faced with low SWB due to unsafe learning environments, family structure, financial distress, social structures, and other structural disparities [9, 11]. AYAs with lower SSS are more susceptible to mental and physical health problems [27, 32]. Generally, AYAs growing up in a sound socio-economic stable home and school environments with clearly defined norms and values are more likely to develop high SSS and SoC compared with their peers in deprived situations, which could influence their SWB [28]. This study is novel in its entirety and seeks to establish the complex relationship (conditional indirect effects) between SSS and SWB by examining other relevant variables like SoC and MR among the secondary school population, which comprised adolescents and young adults. Particularly, this study contributes to the African literature in psychology and related fields and disciplines, situating the research context in more deprived communities in the Northern zone of Ghana. This study is relevant in terms of providing insights into the dynamic relationships among SSS, SWB, SoC and MR within the Ghanaian secondary school context [75–79].

**Fig. 1:**
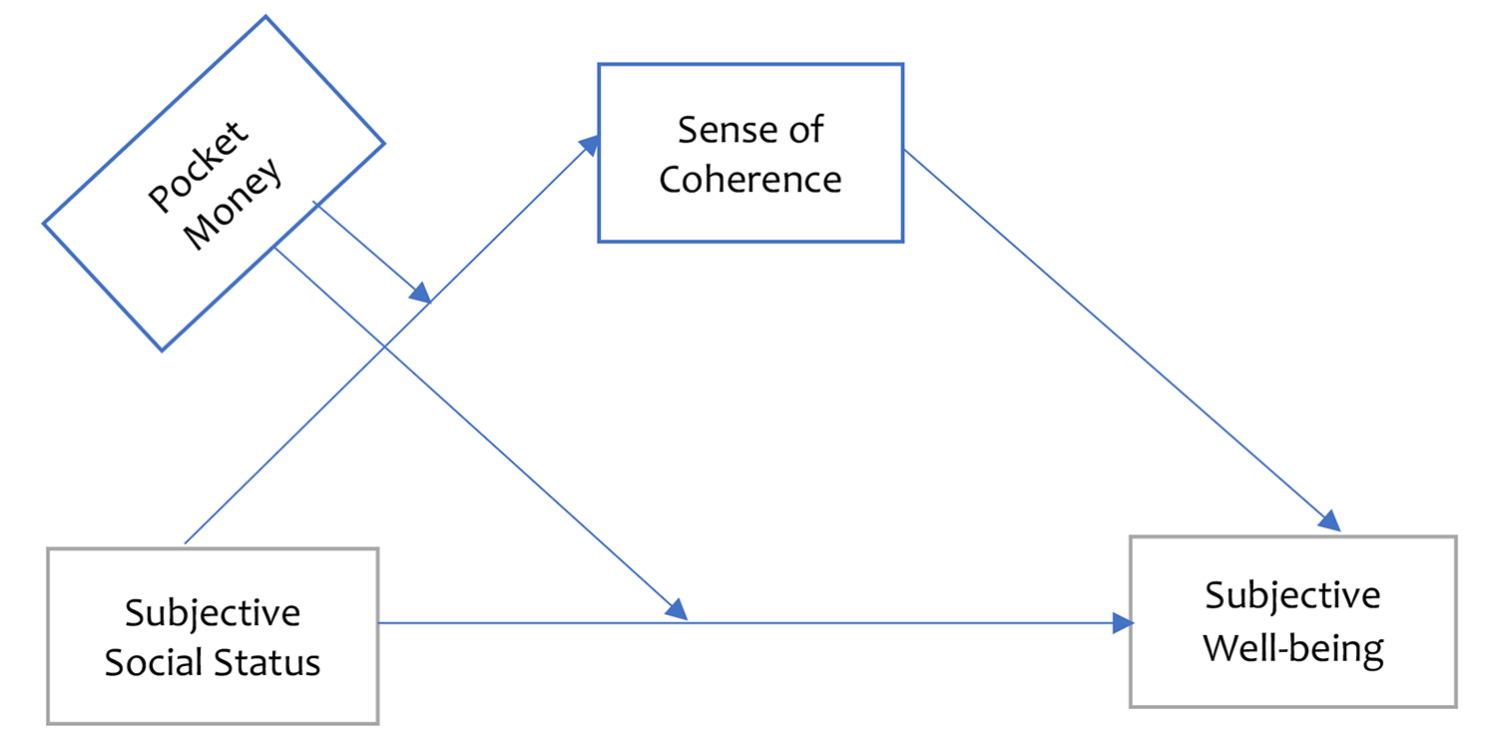
Conceptual Framework Showing the Moderated Mediation Effect of MR and SoC in the Relationship between SSS and SWB.

Ghana’s, culture values interdependence (i.e., collectivism), hence, AYAs may place greater emphasis on visible, agreed-upon markers of status (i.e., objective assessments of social status) as compared to individualistic cultures (i.e., culture valuing independence in countries like Germany, US, Australia) where AYAs greatly valueemphasize their internal thoughts and feelings (i.e., subjective assessments of social status) [78]. AYAs may also emphasize their SSS when there is adequate MR, which may affect how they perceive their social status compared to others. It may also influence their internal resources during stressful situations, which in turn, could affect their SWB. The interrelationships among the key variables in the study, as aforementioned, have been depicted in a conceptual framework (see Fig. 1). Based on the relationships identified, we examined the conditional indirect effect of SSS on SWB through SoC (at different levels of MR) of AYAs in Northern Ghana. Specifically, we hypothesized that: (1) students’ MR will significantly moderate the relationship between SSS and (a) SWB and (b) SoC and (2) SoC will significantly mediate the relationship between SSS and SWB as moderated by the sufficiency of MR.

This study provides a useful discussion on the mental health of AYAs in schools in Northern Ghana, from the lenses of monetary resources, developing resilience and subjective social status. The study would offer institutional administrators an opportunity to find novel means of providing resources needed to enhance the SWB of students to help mitigate potentially adverse health outcomes. The study would provide insights to several stakeholders in education and health promotion, including psychologists, school counsellors and parents on the need to empower AYAs to facilitate quality SWB among AYAs in schools.

## Materials and methods

### Study context

Given that the study’s focus was on adolescents and young adults, we projected that the senior high school setting was more likely to provide a hub for the data to be collected. Thus, this research was conducted in senior high schools (SHS) (formerly known as secondary school) which currently takes students 3 years to complete after their basic education [80]. Public SHS in the Northern part of Ghana were the focus of this research due to the unique characteristics of the populace in such context. Statistics from the World Bank report have revealed that Northern Ghana is a poverty-prone area such that people in such areas have very little chance of breaking out of poverty [81]. It is not surprising that the region is characterized by a higher rate of school drop-outs or increased delayed school access [82]. The region is also noted for having students within their young adulthood stage still at the pre-tertiary level. This study sampled adolescents and young adults (AYAs) who were still in school at the time of data collection.

### Participants’ selection

The study was conducted as part of the global COVID-HL network (www.covid-hl.org). The investigators utilised the network’s instrument to collect primary data (in the year 2021) to address the hypotheses raised. The overall aim of the data collection was to understand the state of digital health literacy, SSS, SoC, SWB and MR and how these variables are related in adolescents and young adults in deprived areas. Using a stratified sampling method, 1096 SHS students (i.e., 91% response rate of 1200 original sample) were selected from the population of SHS students from two regions in Ghana’s Northern zone (i.e., Upper West and Savannah Regions). The two regions were randomly selected after which 5 schools (all mixed-gender schools) were conveniently selected from each region’s capital. In each school, a sample was purposefully obtained to participate in the study.

The participants’ gender distribution were male (*n =* 580, 52.9%), female (*n =* 483, 44.1%) and diverse (*n =* 33, 3.0%). The diverse represents those students who felt they could not be assigned to either the male or female gender category. A greater proportion of the students were in their third year (*n =* 756, 69.0%) with just a few in the first year (*n =* 27, 2.5%). Most of the participants reported they were within the 18–21 years’ range (*n =* 603, 55.0%), while those in the 22–25 years’ range (*n =* 66, 6.0%) were the minority group. Additionally, the majority of the students indicated that they received monetary resource support for their schooling (*n =* 716, 65.3%). However, their monetary resource was less sufficient (*n =* 320, 29.2%). The key criteria for eligibility were that the respondent should be officially enrolled in a secondary school and resided within the Northern parts of the country at the time of the data collection. The participants were required to be within the age brackets of 10 to 25 years. However, the actual age range after data collection was from 14 to 25 years. It must be noted that although the specified age range was used as eligibility, there was no record of a student whose age fell outside the age range of 10 to 25 years.

### Measures

#### Predictor variable: ***subjective social status (SSS)***

McArthur’s Scale of SSS [83] is a single question that permits people to rank their self-perceived socioeconomic standing on a “social ladder” [84]. The scores range from 1 to 10 with higher scores suggesting higher SSS. The respondent is asked: “At the top of the ladder are the people who are the best off—those who have the most money, the most education, and the most respected jobs. At the bottom are the people who are the worst off—who have the least money, least education, and the least respected jobs or no jobs.” Participants were requested to indicate the rank (i.e., from 1 to 10) that perfectly depicts their current social standing. The English version of the McArthur scale, as used in this study, is a psychometrically valid and reliable measure of SSS [16, 83, 85].

#### Outcome variable: ***subjective well-being (SWB)***

In examining the participants’ SWB, the 5-item unidimensional World Health Organization (WHO-5) well-being index was used. The scale options range from 0 = “at no time” to 5 = “all of the time”. Some typical items on the scale include “I have felt active and vigorous”, “I have felt cheerful and in good spirit” and “I woke up feeling fresh and rested”. Each participant’s score is measured based on their responses. After calculating a sum score for each participant, the sum was multiplied by 4 to obtain a composite score ranging from 0 to 100. In screening for depression, lower scores (i.e., < 50) suggest depressive symptoms while higher scores (> 50) reflect sufficient well-being of the participant. Within the Ghanaian setting, Quansah et al. [78] have found the WHO-5 well-being index to be an appropriate instrument for screening depressionwith fair divergent and convergent validity estimates. Using the McDonald Omega method, the data for this scale in this study yielded a reliability estimate of 0.849.

#### Mediator variable: ***sense of coherence (SoC)***

Sense of coherence (SoC), as experienced by participants, was measured with a 9-item scale which had been previously used to measure work-related SoC and developed by Vogt et al. [86]. In examining the current living situation during the COVID-19 era, the 9-item scale was adopted. The instrument comprised three main subscales: comprehensibility (4 items), manageability (2 items) and meaningfulness (3 items) and was measured using a 7-point Likert scale with a range of 0 to 6. Leung et al. [87] noted a Cronbach alpha coefficient value of 0.86 for the scale. However, this study obtained an Omega ω reliability estimate of 0.81. Higher scores on the SoC scale suggest a sufficient SoC whereas a lower score denotes a lower magnitude of SoC.

#### Moderator variable: ***monetary resource***

Monetary resource (MR) is conceptualized as money at students’ disposal. This MR may include school fees or money for buying food and school-related materials and could be given to students by parents/guardians, siblings, family relatives, teachers, friends or even money they have earned through work (i.e., money they have worked for) [53–58]. The participants were asked to rate the sufficiency of this monetary resource at their disposal. The participants were asked “How sufficient do you consider the money at your disposal” with the following response options: “not sufficient”, “less sufficient”, “sufficient” and “completely sufficient”. The participants were required to select the response that best fits them. The investigator’s decision to use these response categories was based on the conviction that since monetary resource extends to any money at disposal (e.g., money for school fees, learning materials, payment of classes), it would be impractical and close to an impossibility to have students without any money at their disposal. Although the level of measurement of students’ MR was supported by Chun et al. [88], we acknowledge that some scholars would have a different viewpoint.

#### Covariates

The study controlled for three key demographic variables by using them as covariates in the analysis. These variables include gender, class (school year level) and age. Dummy variables were created out of the gender and class variables. For gender, the female category was used as the reference group. Similarly, the third-year category was considered the reference group for the class variable. Age was used as a continuous variable.

### Procedure

After ethical approval was obtained, approvals were further obtained from the headmasters and regional education offices of the participating schools by sending official letters before the data collection. After satisfactorily obtaining all approvals, 10 research assistants, who were also indigenes of the two selected regions, were recruited and trained to assist in the data collection. These research assistants had substantial experience in data collection and had a minimum of a bachelor’s degree in the fields of psychology, measurement and evaluation, public health and health education. Some of them also had a Master’s degree as their highest academic qualification. The research assistants were trained on the purpose of the study and how to administer the questionnaire. Also, the researchers, together with the research assistants established a good rapport with the staff and students of the participating schools. As part of the rapport establishment, the researchers explained the study’s purpose to the students and the staff members. The questionnaire was designed and administered in the English Language because all the students could read and write. Aside from ensuring that all COVID-19 safety protocols have been adhered to, ethical considerations such as anonymity and confidentiality were also maintained. The students were asked not to write their names on the questionnaire, and they were assured that their identity would not be revealed to anybody. Further, they were assured that any data provided would be used solely for academic purposes. The selected students were asked to sign the informed consent form. The research assistants distributed the survey instruments to the students who had agreed to participate in the study during the free periods on the schools’ timetables. The items on the questionnaire were explained to the respondents to avoid any misinterpretation. Participants spent approximately 15 minutes responding to the survey instrument after which all the answered questionnaires were collected, sealed in brown envelopes and handed to one of the researchers for safekeeping.

### Statistical analyses

The data were processed with SPSS (version 25, International Business Machines (IBM) Incorporation, New York). The PROCESS macro program complemented the conduct of the analyses. First, descriptive statistics (i.e., frequency, percentage, mean, standard deviation, skewness, kurtosis, maximum and minimum) were conducted to present the demographic variables and explore the key variables. Further, bivariate correlational analyses (i.e., Pearson Product Moment Correlation and Kendall’s Tau-b) were performed to understand the associations among the variables. The moderated mediation analysis was used as the statistical procedure to test the hypotheses in this study. This major analysis was conducted after the variables were screened for possible data entry errors and outliers.

The moderated mediation analysis, also known as conditional process analysis, was performed using Model 8 within the framework of Hayes moderation, mediation and conditional indirect effect analyses plan [89]. A moderated mediation effect implies that the indirect relationship between a predictor (SSS) and a criterion variable (SWB) is explained by a mediator variable (SoC) at different levels of a moderator (MR). Model 8 comprises a variable (i.e., adequacy of MR) moderating the relationship between (a) X (i.e., SSS) and the mediator (i.e., SoC) and (b) X (i.e., SSS) and Y (i.e., SWB). The final aspect of the conditional process analysis entails the effect of SSS on SWB via SoC at different levels of MR sufficiency. The “not sufficient” category was used as the reference group for the analysis. The analysis was conducted at 95% confidence interval and a significance level of 0.05. To assess whether the result was significant or not, the confidence interval was used as the reference point. For a result to be significant, the confidence interval for the parameter should not include zero. The conditional indirect effect analysis was conducted using bootstrapping, specifically with 10,000 bootstrap samples. Using bootstrapping in the analysis maximizes the accuracy of the estimated parameters by providing a straightforward approach to the estimation of confidence intervals and standard errors.

## Results

### Socio-demographic characteristics of participants

The demographic information of the participants was surveyed, including gender, class and age. Other socio-economic data obtained comprise issues about who is funding their schooling activities and whether MR is sufficient. See Table 1 for the distribution of responses from the participants.

**Table 1:** Socio-demographic Characteristics of Participants

Variables	Levels	Counts	Percent
Gender	Male	580	52.9
	Female	483	44.1
	Diverse	33	3.0
Class	First year	27	2.5
	Second year	313	28.5
	Third year	756	69.0
Age	14–17 years	427	39.0
	18–21 years	603	55.0
	22–25 years	66	6.0
Who is funding your schooling?	Support by parents	716	65.3
	Student grant	89	8.1
	Employment within the term	192	17.5
	Employment during term break	63	5.8
	Scholarship	36	3.3
How sufficient is your MR?	Completely sufficient	205	18.7
	Sufficient	252	23.0
	Less sufficient	320	29.2
	Not Sufficient	319	29.1

The majority of the students who participated in the study were females (52.9%) with 44.1% being males and 3% being diverse. More than two-thirds of the students were third year students with very few being in their first year (2.5%) (see Table 1). Further, more than half of the participants were between the ages of 18–21 years. Quite a number of the participants reported age ranges from 14 to 17 years (39%). Regarding who is funding schooling activities, a larger proportion of the students relied on parents (65.3%) and very few were on scholarships (3.3%). Other means of funding schooling activities were student grants (8.1%), employment within the term and employment during the term break.

### Bivariate and descriptive analyses on subjective social status, monetary resource, sense of coherence, and subjective wellbeing

We explored the linear relationships existing among the variables and other descriptive statistics for the variables (see Table 2).

**Table 2:** Bivariate and Descriptive Analyses of the Key Variables

Variables	SSS	SoC	SWB
Subjective Social Status (SSS)	1		
Sense of Coherence (SoC)	0.506^**^	1	
Subjective Well-being (SWB)	0.544^**^	0.596^**^	1
Sufficiency of MR	0.350^**^	0.418^**^	0.497^**^
Mean	3.64	23.95	43.19
Standard Deviation	2.63	10.03	26.66
Skewness	0.792	1.85	0.324
Kurtosis	− 0.319	0.978	− 0.777
Minimum	1	9	00
Maximum	10	63	100

**. Correlation is significant at the 0.01 level (2-tailed).

The output shown in Table 2 shows that there is a positive interrelationship existing among the four variables. These associations ranged from 0.350 to 0.596. For example, SSS was positively associated with SoC, SWB and MR. Similarly, SoC was also positively correlated with SWB and MR. The SSS which ranged from 1 to 10 had a mean of 3.64 and a standard deviation of 2.63. Mean values of 43.19 and 23.95 were obtained for SWB and SoC variables respectively. All the skewness (± 2) and kurtosis (± 7) values were acceptable and depicted that the residuals for the responses on each variable were normally distributed [90, 91].

### Moderating role of monetary resource in the link between subjective social status and (a) subjective well-being and (b) sense of coherence

The study hypothesized that students’ MR will significantly moderate the relationship between SSS and (a) SWB, and (b) SoC. The outcome of the analysis is presented in Table 3.

It is important to mention that the results from the conditional process model confirmed the earlier positive relationship between SSS and SWB (see Table 3). As presented in Table 3, students’ MR significantly moderated the relationship between SSS and SWB, R^2^∆=0.190, *F*(3, 1082) = 7.421; *p <* .001. Thus, the adequacy of students’ MR interacted with the SSS to affect SWB. The analysis revealed that for those who reported MR as less sufficient, a non-significant association was found between SSS and SWB, *B=*-1.173, Boot*SE =* 0.915, Boot*CI*(2.786, 0.622). In contrast, for students who indicated that their MR was completely sufficient, a positive relationship was found between SSS and SWB, *B =* 3.991, Boot*SE =* 0.870, Boot*CI*(2.285, 5.697). Further results in Table 3 also showed that whereas the regression estimate for those in the “not sufficient MR” group was negative, that of those in the “completely sufficient MR” group was positive. A peculiar observation is that when SSS is low or moderate, students with less sufficient MR (*t=*-3.927) are more likely to exhibit higher levels of SWB. However, as the increasing levels of SSS, students with completely sufficient MR would exhibit higher levels of SWB as compared to those with sufficient or less sufficient MR (see Table 3).

Further, the analysis, in Table 3, revealed that the extent of adequacy of MR significantly moderated the relationship between SSS and SoC, R^2^∆=0.014, *F*(3, 1083) = 5.676; *p =* .001. Probing the significant interaction effect, it was found that a positive relationship between SSS and SoC exists for those with less sufficient MR, *B =* 0.680, Boot*SE =* 0.240, Boot*CI*(0.210, 1.151) and completely sufficient MR, *B =* 0.848, Boot*SE =* 0.203, Boot*CI*(0.449, 1.247). Thus, students with sufficient MR, compared with those reporting insufficient MR, exhibited higher levels of SoC, even with the same level of SSS. Thus, irrespective of the level of SSS, those with sufficient MR have higher chances of showing better SoC.

**Table 3:** Moderation Effects of MR in the Relation between SSS and (a) SWB and (b) SoC

Criterion		*B*	Boot SE	*t*	Boot LLCI	Boot ULCI	Conditional effects of the focal predictor					
							MR Levels	B	BootSE	t	Boot LLCI	Boot ULCI
1. SWB*	Constant	33.526	7.907	4.240	18.012	49.041	Not sufficient	0.317	0.543	0.583	− 0.749	1.383
	SSS	1.486	0.592	2.511	0.325	2.648	Less sufficient	-2.505	0.638	-3.927	-3.756	-1.253
	SoC	0.563	0.081	6.996	0.405	0.721	Sufficient	0.313	0.697	0.449	-1.055	1.681
	W1	22.627	3.907	5.791	14.960	30.294	Completely sufficient	1.486	0.592	2.511	0.325	2.648
	W2	7.699	4.033	1.909	− 0.214	15.612						
	W3	9.443	3.393	2.783	2.786	16.099						
	SSS*W1	-1.173	0.915	-1.282	-2.969	0.622						
	SSS*W2	-1.169	0.796	-1.469	-2.731	0.392						
	SSS*W3	3.991	0.870	4.589	2.285	5.697						
	Year 3	Reference group for level										
	Year 1	3.634	5.299	0.686	-6.765	14.032						
	Year 2	2.264	1.843	1.229	-1.352	5.881						
	Female	Reference group for gender										
	Male	-1.249	1.700	− 0.734	-4.585	2.088						
	Diverse	-9.648	4.923	-1.960	-19.308	0.012						
	Age	− 0.645	0.396	-1.628	-1.422	0.132						
2. SoC**	Constant	15.206	2.948	5.158	9.422	20.990	Not sufficient	− 0.201	0.223	− 0.902	− 0.640	0.237
	SSS	− 0.201	0.223	− 0.902	− 0.640	0.237	Less sufficient	0.680	0.240	2.836	0.210	1.151
	W1	-2.208	1.473	-1.499	-5.099	0.682	Sufficient	− 0.082	0.263	− 0.310	− 0.598	0.435
	W2	-1.767	1.521	-1.162	-4.751	1.217	Completely sufficient	0.848	0.203	4.170	0.449	1.247
	W3	-2.470	1.278	-1.933	-4.978	0.038						
	SSS*W1	0.882	0.327	2.695	0.240	1.523						
	SSS*W2	0.120	0.345	0.347	− 0.558	0.797						
	SSS*W3	1.049	0.299	3.513	0.463	1.636						
	Year 3	Reference group for level										
	Year 1	-5.919	1.992	-2.972	-9.827	-2.011						
	Year 2	− 0.414	0.695	− 0.596	-1.779	0.950						
	Female	Reference group for gender										
	Male	-1.597	0.640	-2.496	-2.853	− 0.342						
	Diverse	7.385	1.844	4.004	3.766	11.004						
	Age	0.520	0.149	3.496	0.228	0.811						

*Model summary: R^2^ = 0.291, *F*(13, 1082) = 7.698, *p <* .001; *R*^2^∆=0.190, *F*(3, 1082) = 7.421; *p <* .001.

**Model summary: R^2^ = 0.280, *F*(12, 1083) = 7.662, *p <* .001; R^2^∆=0.014, *F*(3, 1083) = 5.676; *p =* .001.

W1- Less sufficient, W2- Sufficient, W3- Completely sufficient, Reference group – not sufficient.

Note: BootLLCI – Bootstrap Lower Limit Confidence Interval, BootULCI – Bootstrap Upper Limit Confidence Interval, BootSE- Bootstrap Standard Error, t- t-value, F- Model fit indicator for the specified model, B- Unstandardised regression coefficient, R^2^∆ - A change in variance proportion accounted for by the moderator.

### Moderated mediation effect of monetary resource and sense of coherence in the relationship between subjective social status and subjective well-being

The study also hypothesized that SoC will significantly mediate the relationship between SSS and SWB as moderated by the sufficiency of MR variable. The details of the results are shown in Table 4.

**Table 4:** Conditional Direct and Indirect Effects of MR and SoC on SSS and SWB

Conditional Effects	MR Levels	B	BootSE	LLCI	ULCI
Direct effect(s) of X on Y:	Not sufficient	-2.505	0.638	-3.927	-3.756
	Less sufficient	0.313	0.697	0.449	-1.055
	Sufficient	0.317	0.543	0.583	− 0.749
	Completely sufficient	1.486	0.592	2.511	0.325
Indirect effect of X on Y: SSS -> SoC -> SWB	Not sufficient	− 0.113	0.169	− 0.453	0.218
	Less sufficient	− 0.046	0.144	− 0.319	0.247
	Sufficient	0.383	0.161	0.087	0.722
	Completely sufficient	0.478	0.142	0.208	0.765
Index of moderated mediation (difference between conditional indirect effects)	Contrast	Index	BootSE	LLCI	ULCI
	W1	0.067	0.220	− 0.347	0.528
	W2	0.497	0.236	0.069	0.990
	W3	0.591	0.231	0.154	1.070

X - Subjective social status; Y – Subjective well-being

W1- Less sufficient, W2- Sufficient, W3- Completely sufficient; Comparison group: Not sufficient

D_index_ - the difference between conditional indirect effects.

The analysis showed a significant negative direct effect of SSS on SWB only for those who had insufficient MR, *B=*-2.505, Boot*SE =* 0.638, Boot*CI*(-3.927, -3.756) whereas a significant positive direct effect of SSS on SWB was discovered for those who had completely sufficient MR at school, *B =* 1.486, Boot*SE =* 0.592, Boot*CI*(2.511, 0.325) (see Table 4). The results further highlighted that SoC significantly explained the relationship between SSS and SWB for those who had sufficient MR, *B =* 0.383, Boot*SE =* 0.161, Boot*CI*(0.087, 0.722), as well as those with sufficient MR, *B =* 0.478, Boot*SE =* 0.142, Boot*CI*(0.208, 0.765).

We further tested for the difference between the indirect effects of SoC in the association between SSS and SWB for students with sufficient MR and those with completely sufficient MR. A significant difference in the indirect effects of SoC (in the relation between SSS and SWB) for students with insufficient MR and those with sufficient MR was revealed, D_index_=0.497, *SE =* 0.236, Boot*CI*(0.069, 0.990). Similarly, significant differences in the indirect effect for students with insufficient MR and those with completely sufficient MR could be found, D_index_=0.591, *SE =* 0.231, Boot*CI*(0.154, 1.070).

### The roles of the control variables in the moderated mediation model

The analyses controlled for three demographic variables; namely, year of study (class), gender and age. Although the focus of the study was not on these control variables, their roles in the analyses are important to guide decisions concerning the selection of control variables in future research as well as help in the replication of the study. It was discovered that students’ level of study, gender and age failed to predict their SWB. However, these demographic factors significantly predicted SoC. For example, first year students compared to third year students exhibited lower levels of SoC, *B=*-5.919, Boot*SE* = 1.992, Boot*CI* (-9.827, -2.011). Female students were also found to exhibit higher levels of SoC compared to their male counterparts, *B=*-1.597, Boot*SE* = 0.640, Boot*CI* (-2.853, − 0.342). Students who reported themselves as diverse gender showed higher levels of SoC compared to male and female students, *B =* 7.385, Boot*SE* = 1.844, Boot*CI* (3.766, 11.004). Older students were found to exhibit higher levels of SoC compared to younger ones, *B =* 0.520, Boot*SE* = 0.149, Boot*CI* (0.228, 0.811).

## Discussion

Having preliminarily established a significant positive association between SSS and SWB and as has also been found in recent studies globally [6, 22, 23, 27, 29, 31, 32], this research addressed the gaps in the literature by further assessing the indirect effect of SoC and MR in this relationship among AYAs in secondary schools in northern Ghana. Based on the argument that the correlation between SSS and SWB is spurious and confounded by other key variables which co-exist in the lives of AYAs [51], two hypotheses were tested: (1) Students’ MR will moderate SSS-SWB relationship as well as the link between SSS and SoC; (2) SoC will significantly mediate the relation between SSS and SWB as moderated by the sufficiency of MR.

The findings of this study showed that students’ MR moderated the link between SSS and SWB. The relationship was negative for those with insufficient MR and positive for those who reported completely sufficient MR. Sufficient evidence was also gathered to support the hypothesis that students’ MR will moderate the relation between SSS and SoC; it was found that students with sufficient MR, compared to those with insufficient MR, exhibited higher levels of SoC, even with the same level of SSS. This finding stresses the significant role of students’ MR in strengthening the SSS association with SoC and SWB. The presence of sufficient MR might have built the confidence and resilience of these students resulting in their increasing efforts [46].

Importantly, AYAs with sufficient MR might feel in control of financial strain as a potential stressor in their lives both in school and/ or perhaps at home or elsewhere. It must be mentioned that economic capital in its entirety plays a huge role in health and other outcomes. This situation may translate into SoC and consequently, AYAs experiencing the feeling that life is going on well and can influence what is happening in their lives [92]. These perceptions might further boost AYAs’ confidence, happiness, and life satisfaction. Scholars have reiterated that students in possession of MR are more likely to learn the value of money, how to manage, develop a sense of responsibility and independence and make life choices [55, 58]. It is not surprising that other scholars have mentioned that the adequacy of MR builds autonomy, responsibility, and decision-making ability among AYAs [92]. For example, students who develop these attributes through the availability of sufficient MR would feel confident in their capability to cope with everyday stressors, although, young people without sufficient MR but report moderate to high SSS might also perceive a high SoC and SWB. For instance, a student confronted with a pandemic like COVID-19 may feel that having sufficient MR to either buy protective equipment and/ or seek better healthcare, may contribute to a high SoC and SWB.

Admittedly, AYAs in schools with sufficient MR are likely to come from families with high socio-economic status or high-income levels as has been revealed in previous research [93]. This observation is quite understandable since the MR usually comes from the incomes of family relatives (e.g., father, mother, siblings, etc.) and thus, a reflection of a higher socio-economic status of the family. Although a large body of knowledge has established this positive link between students’ MR and family income [93], we also acknowledge that, based on cultural reasons or disparities, some wealthy families may not provide sufficient MR to their wards in schools. Given this premise, it is possible that AYAs’ sufficient level of MR may not be directly responsible for building their SoC and improving SWB. For instance, a student (either adolescent or young adult) may have confidence in coping with life challenges or stressors because they are confident that they have adequate resources to handle the stressors they experience. This study provides useful direction in terms of the relationship of students’ MR with SoC and SWB.

A peculiar observation from the study findings is that when SSS is low or moderate, the presence of completely less sufficient MR among AYAs does not greatly enhance SWB, even though those with sufficient MR are more likely to exhibit higher levels of SWB compared to those in the other MR response categories. However, at high levels of SSS, students with completely sufficient MR exhibit higher levels of SWB compared to those with sufficient or less sufficient MR. This finding highlights the essence of a high level of SSS with high sufficient MR in ensuring greater levels of SWB. This finding suggests that even when there is completely sufficient MR for their wards in the absence of high SSS, such students are likely to exhibit some level of depressive symptoms [94]. This finding may be explained by the fact that this study comprised AYAs in schools who are now exploring their identities and building their sense of individual SSS via relating with several students from diverse backgrounds while retaining closely their connection to the SSS of their families [95, 96]. This peculiar outcome suggests that the presence of a high level of SSS and completely sufficient MR may concurrently enhance the SWB of AYAs in schools. This strong relationship between MR and SSS is justified based on the assumption that having sufficient MR can help build one’s SSS, especially, when individuals base their SSS on their MR relative to their peers in schools. For example, in-school AYAs who have sufficient MR to purchase anything of their choice may place themselves on higher status relative to their peers who cannot afford to purchase similar items. The foregoing could explain why MR moderated the SSS relationships with SoC and SWB.

Notably, over 65% of this study’s participants relied on parents/guardians for MR, and given that the study was conducted in a highly poverty-prone setting, AYAs in schools may experience some level of bad feelings knowing well that their parents do not have that kind of money at their disposal. The little financial resources at disposal of the family may be used to cater for the needs of other siblings in the household- this assertion is strongly grounded in the fact that Ghana operates within a collectivist culture. Given this cultural orientation, people do not only cater for their immediate family but may also financially support other external family relatives, close friends and community members [78]. The result, to some extent, shows that those vulnerable groups are disadvantaged already by a low family socio-economic status, culminating in a second level of disadvantage when the parents try to help . This situation is applicable in the context where AYAs in schools do not solely depend on parents or guardians for MR but rely on some jobs they engage in within the academic term or during term breaks, especially when the funds from these jobs are not so sufficient to comfortably cater for their needs.

The study also revealed a significant difference in the indirect effects of SoC (in the relation between SSS and SWB) for students with insufficient MR against those with completely sufficient and sufficient MR. Essentially, SoC significantly explained the relationship between SSS and SWB for those with (completely) sufficient MR and not for those with insufficient or less sufficient MR. The presence of (completely) sufficient MR among AYAs, as earlier indicated, could influence their SSS [59], which would further predict their SoC to explain the variations in their SWB status. For those who reported insufficient or less sufficient MR, it might be very difficult to perceive themselves at a high SSS and this might significantly lead to perhaps non-existent or very little ability to cope and improve their SWB. Students in this category may adopt maladaptive coping strategies (such as behaviour disengagement coping strategy or avoidance coping style) to cope with life stressors. Thus, this claim could justify why SoC could not explain the link between SSS and SWB for the group with insufficient or less sufficient MR. Similarly, the reliance of AYAs on religious coping strategies to cope with everyday life stressors may result in a limited role played by SoC in the SSS/SWB relationship. The finding implies that SoC should not be interpreted as an autonomous personal coping resource contributing to the favourable development of AYAs’ well-being, but as a factor that could work in connection with other available strategies, especially in the absence of sufficient MR. Other scholars who could not establish the intervening role of SoC in the socio-economic status-health relationship [61] have supported this view.

The overall understanding of the moderated mediation result supports Antonovsky’s salutogenic theory which illustrates the interrelationship between SoC, life experiences, GRR and health outcomes [34–36]. Operating within the theory’s framework, it can be argued that AYAs movement along the health outcome continuum is explained by the stressors they experience in their lives and how they cope with these stressors using existing resources or searching for other resources. This study’s findings showed that for AYAs to exhibit either poor or better SWB depends on successfully coping with everyday life stressors by drawing on their SSS, MR and SoC. As postulated by the salutogenic theory and found in this study, AYAs who had completely sufficient MR at their disposal, high SSS and high level of SoC are more likely to have better SWB. However, those AYAs with no or less sufficient GRRs (i.e., insufficient or less sufficient MR, low SSS, and low SoC) would rather move towards poor SWB. The non-existent of GRRs or inability to successfully apply appropriate coping could generate mental health challenges (e.g., anxiety, depression, sleeping disturbance, stress) and poor SWB. These views are consistent with the arguments put forward by Super et al. [97] in explaining how individuals move towards a health ease/dis-ease continuum.

### Strengths and limitations

This research draws its strength through the application of a more robust statistical procedure (i.e., conditional process analysis) to investigate the complex relationship existing among SSS, MR, and SoC to explain the variabilities in SWB among AYAs in secondary schools. The research findings provide solid support to theory (i.e., Salutogenic theory) to underscore that the relation between SSS and SWB is not a straightforward one; but requires other intervening variables. This insight helps provide useful information for improving the SWB of AYAs in schools. Despite the strengths, the study is not without limitations. The use of a cross-sectional survey design makes it impossible to assume causal relationships among the variables. Therefore, longitudinal, and experimental studies would provide a much better view of the causal link among the variables. Adopting the self-report measures may affect the validity of responses when recalled responses are not accurate. Only AYAs in secondary schools were recruited for the study and this limits the generalization of the results. Future studies are encouraged to conduct similar studies among AYAs who have dropped out of school or have not attended school before. Cultural-related variables such as ethnicity, region/district and religion were not considered or controlled in this inquiry, accordingly, future studies should control for these variables.

### Practical implications

The findings of this research provide deep understanding on improving the SWB of AYAs in secondary schools through empowerment and reflection on activities related to health promotion. This intervention is important as such activities related to improving SWB could also promote increasing academic efforts [98], academic performance, school adjustment and more effective studying habits [99]. The government of Ghana should direct their efforts towards designing policies that create enabling environments for parents/guardians to secure occupation with higher wages, which would, in turn, provide sufficient MR for their children and help reduce education-related inequities.

In the absence of sufficient MR, there should be social interventions (e.g., loan schemes, bursaries) in schools for students with inadequate MR. According to the Salutogenic model, resources like SoC exist within the individual and as such, intervention programs can successfully imbibe in people with high levels of SoC and SWB as has been established in previous research [100, 101]. The findings of this study not only point to rolling out intervention programs to AYAs in schools but also focus on empowering these young individuals to mobilize and reflect on the limited resources available to them. Empowering AYAs might offer a state of balance for them by reflecting on how to use existing resources to move towards better SWB. The study calls on several stakeholders in education and health promotion including psychologists, school counsellors and parents to be involved in the training and empowering process to facilitate superior SWB among AYAs in schools.

## Conclusion

The findings from the current research underscore the need for providing sufficient to completely adequate MR for students in secondary schools in Ghana, especially those from vulnerable populations. This intervention is necessary to improve the SWB and SoC of these students. Interestingly, the perception of the students on their social status is largely a relevant variable in this process. The findings of this research also place much emphasis on SoC as key in explaining the relationship between SSS and SWB; however, this role of SoC differs depending on the extent of MR adequacy. That is, SoC is more potent in improving SWB when there is the presence of sufficient/completely sufficient MR and a high level of subjective social status. However, low to moderate SSS with less sufficient to sufficient MR does not result in better SWB, reflecting the issue of how inequalities generate further inequalities and thus, contribute to social inequities in general.

## Data Availability

Anonymized data is available upon reasonable request through the corresponding author.

## Declarations

### Competing of interests

None declared.
